# Co-Expression Networks Unveiled Long Non-Coding RNAs as Molecular Targets of Drugs Used to Treat Bipolar Disorder

**DOI:** 10.3389/fphar.2022.873271

**Published:** 2022-04-08

**Authors:** Trang TT. Truong, Chiara C. Bortolasci, Briana Spolding, Bruna Panizzutti, Zoe SJ. Liu, Srisaiyini Kidnapillai, Mark Richardson, Laura Gray, Craig M. Smith, Olivia M. Dean, Jee Hyun Kim, Michael Berk, Ken Walder

**Affiliations:** ^1^ School of Medicine, IMPACT, Institute for Innovation in Physical and Mental health and Clinical Translation, Deakin University, Geelong, VIC, Australia; ^2^ Genomics Centre, School of Life and Environmental Sciences, Deakin University, Burwood, VIC, Australia; ^3^ The Florey Institute of Neuroscience and Mental Health, University of Melbourne, Parkville, VIC, Australia; ^4^ Orygen, The National Centre of Excellence in Youth Mental Health, Centre for Youth Mental Health, Florey Institute for Neuroscience and Mental Health and the Department of Psychiatry, The University of Melbourne, Melbourne, VIC, Australia

**Keywords:** bipolar disorders, co-expression network, WGCNA, mood stabilizers, lncRNAs, treatments, mood disorders

## Abstract

Long non-coding RNAs (lncRNAs) may play a role in psychiatric diseases including bipolar disorder (BD). We investigated mRNA-lncRNA co-expression patterns in neuronal-like cells treated with widely prescribed BD medications. The aim was to unveil insights into the complex mechanisms of BD medications and highlight potential targets for new drug development. Human neuronal-like (NT2-N) cells were treated with either lamotrigine, lithium, quetiapine, valproate or vehicle for 24 h. Genome-wide mRNA expression was quantified for weighted gene co-expression network analysis (WGCNA) to correlate the expression levels of mRNAs with lncRNAs. Functional enrichment analysis and hub lncRNA identification was conducted on key co-expressed modules associated with the drug response. We constructed lncRNA-mRNA co-expression networks and identified key modules underlying these treatments, as well as their enriched biological functions. Processes enriched in key modules included synaptic vesicle cycle, endoplasmic reticulum-related functions and neurodevelopment. Several lncRNAs such as *GAS6-AS1* and *MIR100HG* were highlighted as driver genes of key modules. Our study demonstrates the key role of lncRNAs in the mechanism(s) of action of BD drugs. Several lncRNAs have been suggested as major regulators of medication effects and are worthy of further investigation as novel drug targets to treat BD.

## Introduction

Bipolar disorder (BD) is among the top 10 causes of disability globally with potentially devastating consequences for individuals as well as wider society (Association, 2013; James et al., 2018). Although pharmacotherapy is the first-line management for BD, suboptimal outcomes and treatment-resistance are common, and polypharmacy is often required (Garnham et al., 2007; Kukreja et al., 2013; Fornaro et al., 2016). Despite the pressing demand for new therapeutic agents, new drug discovery for BD remains stagnant due to our lack of understanding of both the underlying pathophysiology of the disorder and the mechanism(s) of action of currently available drugs.

Studies have suggested a role for dysregulation of gene expression in the pathophysiology of BD, not only with protein-coding but also with non-coding RNAs (Ponting et al., 2009; Zuo et al., 2016; Luykx et al., 2019). Most of the non-coding transcriptome is made up of long non-coding RNAs (lncRNAs). These comprise >200 nucleotides, and are key regulators of gene expression possibly via epigenetic regulation and chromatin remodelling (Hrdlickova et al., 2014). LncRNAs are highly expressed in the brain, and they may contribute to the development of psychiatric diseases including BD (Qureshi et al., 2010; Zuo et al., 2016; Bella and Campo, 2021). In a genome-wide association study (GWAS), Hou *et al.* found a significant association between lithium response and four single nucleotide polymorphisms (SNPs) in a region containing two lncRNAs, suggesting a role for these lncRNAs in the biological mechanism(s) of lithium (Hou et al., 2016). Given that little is known regarding how lncRNAs may be involved in the mechanism(s) of action of drugs used to treat BD, comprehensive gene expression analysis that includes lncRNAs will help to elucidate the transcriptional perturbations underlying the therapeutic effects.

When researching brain disorders, reductionist approaches are still dominant: individual candidate receptors or genes are often analysed singly, disregarding their interaction with other molecular factors. To mitigate such limitation, network-based approaches that consider modules of genes as key regulators offer a complementary method to address the complex dysregulation occurring in neuropsychiatric conditions such as BD (Gaiteri et al., 2014). Moreover, it remains unclear how most genes function, which has become more puzzling with the recent identification of novel non-coding genes. A detailed global view of genome-wide transcriptional perturbations can be yielded with the advent of high-throughput sequencing technologies. Genes with functional linkages tend to be co-expressed across various biological states, and co-expression networks yielded from RNA-sequencing data can infer gene function and molecular mechanisms by associating genes of unknown function with biological processes (Gaiteri et al., 2014; Okamura et al., 2015).

In the current study, we investigated the mechanism(s) of action of four widely prescribed BD drugs (i.e., lamotrigine, lithium, quetiapine and valproate) using network-based approaches, with an emphasis on the role of lncRNAs. We hypothesised that mRNA-lncRNA crosstalk and regulatory patterns in neuronal cell models will unveil insights into the complex mechanisms of BD drugs and highlight potential new targets for drug development.

## Materials and Methods

**FIGURE 1:**
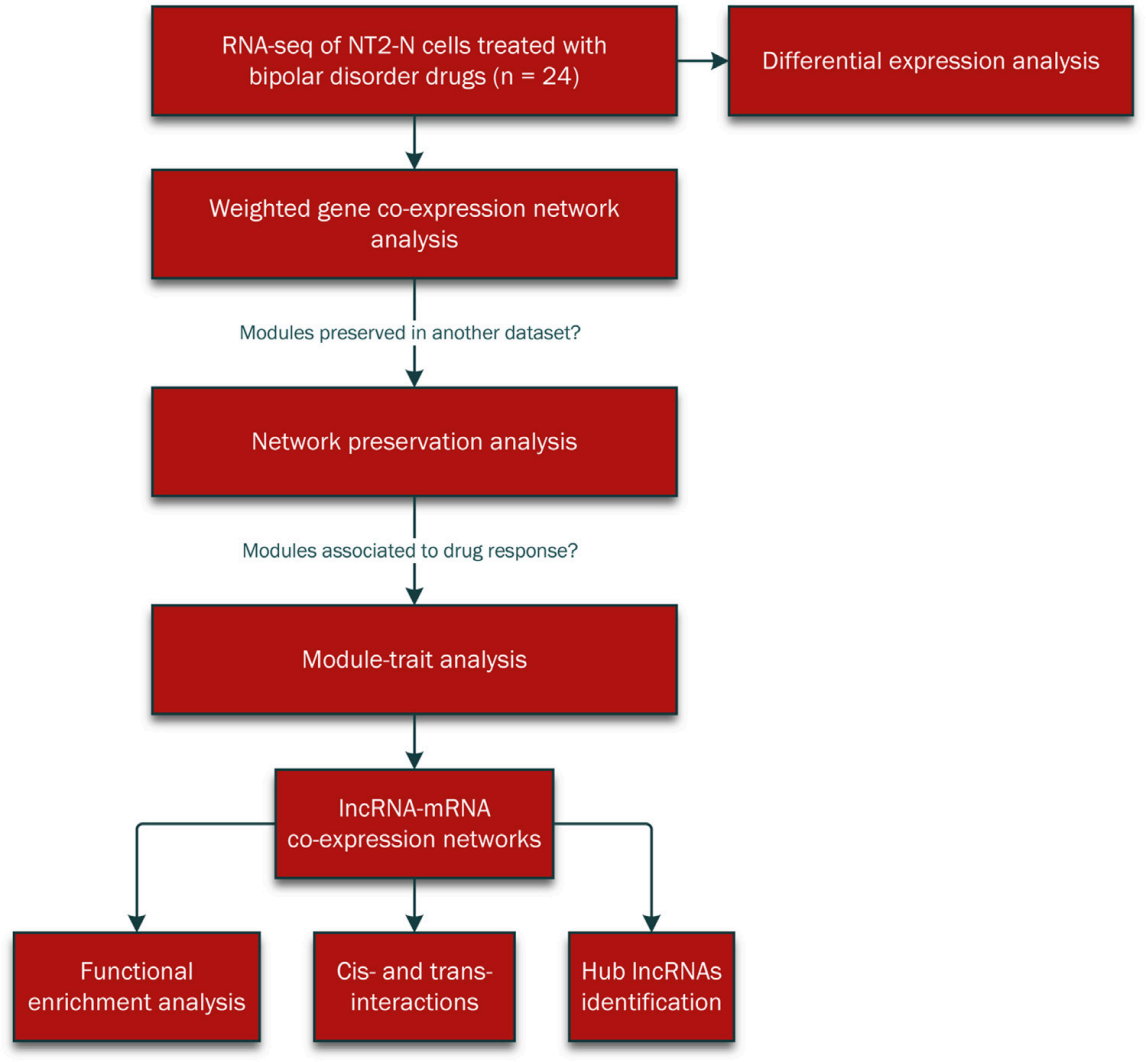
The methodology workflow the current study.

The summary flowchart of the methodology was demonstrated in Figure 1.

### Cell Culture

For the model of human neurons, the study used NT2 human teratocarcinoma cells (CVCL_0034, ATCC, Manassas, VI, United States), differentiated into post-mitotic neuronal cells (NT2-N) after treatment with retinoic acid (Pleasure et al., 1992; Megiorni et al., 2005). Cells were cultured and differentiated as previously described (Bortolasci et al., 2018). Concisely, NT2 cells were maintained in Dulbecco’s modified Eagle’s Medium (DMEM; Life Technologies, Melbourne, Australia), 10% fetal bovine serum (FBS; Thermo Fisher Scientific, Melbourne, Australia) and 1% antibiotic-antimycotic solution (Life Technologies). NT2-N cells were induced from NT2 cell cultures by treating with 10^−5^ M retinoic acid (Sigma-Aldrich, Sydney, Australia) for 28 days with media refreshed every 2–3 days. For experiments, cells were seeded onto 24-well plates coated with 10 μg/ml poly-d-lysine (Sigma-Aldrich) and 10 μg/ml laminin (Sigma-Aldrich) at 2 × 10^5^ cells/well with further addition of mitotic inhibitors (1 µM cytosine and 10 µM uridine; Sigma-Aldrich) for a total of 7 days, and the media was refreshed every 2–3 days to generate an enriched culture of differentiated neuronal cells (NT2-N). *NeuroD*, *Tau*, and *GluR* expression levels in differentiated cells were measured to verify the effectiveness of the differentiation process (data not shown).

### Drug Treatments

NT2-N cells were treated with lamotrigine (50 µM), lithium (2.5 mM), quetiapine (50 µM), or valproate (0.5 mM) for 24 h (4 replicates for each group). All drugs were purchased from Sigma-Aldrich (Sydney, Australia). Vehicle control cells were treated with 0.5% Milli-Q water for lithium or valproate controls, and 0.2% DMSO for lamotrigine or quetiapine controls. The drug doses were chosen based on prior dose response studies in our lab to ensure no impact on cell viability (Kidnapillai et al., 2018). Using these doses, we have previously observed differences in gene expression after 24 h treatment (Bortolasci et al., 2018; Bortolasci et al., 2020b). These drugs have also been demonstrated to alter gene expression levels *in vitro* after 24 h in other studies (Nahman et al., 2012; Meng et al., 2019).

### Genome-Wide Gene Expression Measurement

Total RNA was extracted from cells post-treatment using RNeasy^∗^ mini kits (Qiagen, Melbourne, Australia), then checked for quality and quantity using an Agilent 2100 Bioanalyzer (Agilent Technologies, Melbourne, Australia) and NanoDrop 1000 (Thermo Fisher Scientific), respectively. RNA-seq libraries were prepared for all samples from 1 µg total RNA using a TruSeq RNA Sample Preparation Kit (Illumina, Victoria, Australia) as per the manufacturer’s instructions. Samples were analysed on a HiSeq 2500 Rapid system (50 bp single end reads; Illumina) to measure genome wide mRNA expression, which yielded an average of about 10 × 10^6^ reads/sample. The single end sequencing offered sufficient quantification of gene expression levels in well-annotated organisms such as *Homo sapiens* at cheaper cost than the paired-end technology (Conesa et al., 2016).

### Genome-Wide Gene Expression Analysis

The raw data yielded in FASTQ format were aligned to reference genomes using the Deakin Genomics Centre RNA-Seq alignment and expression quantification pipeline (https://github.com/m-richardson/RNASeq_pipe). Briefly, the pipeline included raw read quality filtering and adapter trimming (ILLUMINACLIP:2:30:10:4, SLIDINGWINDOW:5:20, AVGQUAL:20 MINLEN:36) with Trimmomatic v35 (Bolger et al., 2014), and alignment to the reference genome using STAR v2.5 in 2-pass mode (Human genome version GRCh38) (Dobin et al., 2012). Raw reads were deposited at the Gene Expression Omnibus (GEO) database under the accession numbers GSE197966.

For differential abundance testing, gene expression quantified at the gene level was compiled into a m x n matrix from individual sample counts. Genes with low expression were omitted (<1 cpm in *n* samples, where *n* is the number of samples in the smallest group for comparison), and the data was normalized using the weighted trimmed mean of M-values (TMM) using edgeR (Robinson et al., 2009) in R (R Core Team, 2021). EdgeR was used to determine differential gene expression, and the Benjamini–Hochberg method was used to assess false-discovery rate (FDR) adjusted q-values for multiple tests (Benjamini and Hochberg, 1995).

### Weighted Gene Co-Expression Network Analysis

WGCNA is an approach utilizing gene expression data to construct co-expression networks weighted for high correlations (Langfelder and Horvath, 2008) and was used in this study to evaluate correlation between lncRNAs and mRNAs. The RNA-seq data was used as input for the R package WGCNA (Langfelder and Horvath, 2008; R Core Team, 2021), from which a pairwise bi-weight mid-correlation matrix was computed and then transformed into an adjacency matrix. To construct a scale-free network, each absolute mid-correlation value was raised to a soft-thresholding power. Soft-thresholding amplified disparity between strong and weak correlations, leading to the construction of the scale-free network. We chose power 7, which was the lowest power for which the scale-free topology fit index reached 0.8. To minimise the spurious connections, WGCNA utilised the topological overlap measure (TOM) accounting for how large the overlap of each gene pair’s network neighbours. The TOM matrix considered as a similarity measure was then transformed into pairwise dissimilarity measure (calculated by 1-TOM) for the hierarchical clustering of genes. From this, tightly connected genes would be clustered for module assignment (dynamic tree cut algorithm), and unassigned genes with weak connections would not be considered for further analyses. The default value of 0.25 was set as the threshold for cut height to merge possible similar modules. The expression level of each module was represented by an eigengene value. Module membership values were also calculated, which reflect the degree of correlation between genes and modules. Higher absolute values of module memberships mean stronger correlation, while zero values mean no association.

### Module Preservation and Module-Trait Analyses

To validate the reproducibility of key modules, module preservation analysis was undertaken with an independent dataset (Bortolasci et al., 2020a). The most common measure of module preservation recommended by the authors of WGCNA is Zsummary, which considers the density and connectivity patterns of module nodes, as well as the overlap among module membership. Higher Zsummary statistics indicate more highly preserved modules. Notably, Zsummary can be heavily dependent on module sizes, which might bias the statistics towards bigger modules (Langfelder et al., 2011). In our network, module sizes were spread in a wide range from 30 to more than 3000, hence we only used a cut off Zsummary >10 as recommended for strong preservation and then ranked the degree of preservation based on medianRank. The lower medianRank one module has, the stronger preservation it tends to exhibit in another dataset. Langfelder et al. found medianRank to be much less dependent on module sizes, and hence it can be a useful measure for comparing relative preservation among multiple modules (Langfelder et al., 2011).

To identify key modules related to the response to drug treatments, association analysis between a module and the trait of each pairwise comparison group (e.g., lithium–water, quetiapine–DMSO) was performed based on module eigengenes. Student asymptotic *p*-values were calculated for correlation values, and then adjusted for multiple testing with Benjamini–Hochberg false discovery rate correction.

### Functional Enrichment Analysis

In order to find potential pathways that might be driven by key modules, functional enrichment analysis was deployed on all genes from each module using the R package ClusterProfiler (Yu et al., 2012) with pathway reference from the Gene Ontology (GO) database (Gene Ontology, 2004) filtered by “Biological Process”. ClusterProfiler is a popular enrichment package, which was extensively utilized in medical studies (Liu et al., 2021; Liu et al., 2022a; Liu et al., 2022b).

Despite their comprehensiveness, GO terms are prone to redundancy, which might complicate the functional interpretation. Hence, we deployed the Enrichment Map from Cytoscape (Merico et al., 2010) to intuitively visualise the enrichment results in a network-based manner. Enrichment Map clusters similar gene sets together (based on how well-connected their genes are), offering a more concise overview of enriched biological functions. In the map, gene sets were illustrated as nodes and would be linked together if they share overlapping genes. The overlapping metric was calculated based on a combination of Jaccard coefficient and overlap coefficient (50% and 50% respectively), and a cut-off of 0.375 was set to define edges forming between nodes.

### lncRNA-mRNA Co-Expression Networks, Identification of Hub lncRNAs, Cis- and Trans-Interactions

The lncRNA-mRNA co-expression network of key modules associated with the drug treatments was built using the bi-weight mid-correlation of lncRNA-mRNA pairs. The lncRNA-mRNA pairs whose weights of connection were at least 0.15 were selected for co-expression network construction. The network was imported and visualized in Cytoscape (Shannon et al., 2003). To find potential key regulators of each module, hub lncRNAs were identified by degree of centrality levels calculated using Cytoscape (Shannon et al., 2003).

To find potential cis-targets of lncRNAs in each key module, nearest protein coding genes located 100 kb upstream or downstream from the transcription start site of lncRNAs were identified by BEDTools (Quinlan and Hall, 2010) v2.27 with annotation from human genome version GRCh38. Otherwise, the other mRNAs in the module would be considered to have trans-interaction with lncRNAs in the module. Among these trans-interactions, we evaluated if any of them could be due to the regulation of transcription factors on lncRNAs as co-expression relationships can be interpreted in both directions. Hence, we searched for trans-acting transcription factors found nearby lncRNAs (from −30 to 10 kb away from transcription start sites) using chromatin immunoprecipitation followed by sequencing (ChIP-seq) data from the ChIPBase database (Zhou et al., 2017).

## Results

### Differential Expression Analysis

The characteristics of RNA-seq results for each drug treatment and differential analysis mainly for mRNAs have been reported in our previous publication (Bortolasci et al., 2020b). For lncRNAs, we found 1044 were expressed at detectable levels in NT2-N cells. Among them, 282 lncRNAs were differentially expressed (FDR <0.05) following treatment of NT2-N cells with one or more drugs used to treat BD (Supplementary Table S1). While differential expression analysis is the initial approach for evaluating genes driving the difference between different phenotypes (in our case, treatments and controls), it can be challenging to study lncRNAs which tend to be expressed at lower levels than mRNAs (Ulitsky, 2016; 2019). The significance of *p*-values was evaluated based on the whole distribution of all genes detected in our RNA-seq (including mRNAs, lncRNAs), thus any genes with lower fold change would be less likely to be significantly differentially expressed after correction for multiple testing. Therefore, differential expression analysis was complemented with an unsupervised gene co-expression analysis method to discriminate small but coherent patterns of differences.

### Weighted Gene Co-Expression Network Analysis

After identifying individual lncRNAs that were differentially expressed, we next sought to identify co-expressed networks of genes from the whole transcriptome, as this approach may reveal additional lncRNAs of interest and other important mechanistic insights. Without any *a priori* defined groups like in differential expression analysis, weighted gene co-expression network analysis (WGCNA) on the whole transcriptome explored highly correlated and consistent transcriptional patterns across 24 samples of our RNA-seq results (four samples x two vehicles, four samples x four drug treatments). While bigger sample size would be more ideal, 24 samples are within the recommendation of the WGCNA workflow for robust analyses (Langfelder and Horvath, 2022). WGCNA identified 29 modules and the full list of genes in each module is shown in Supplementary Table S2.

To validate the reproducibility of the modules in the current WGCNA network, we performed preservation analysis of topology and connectivity patterns against an independent RNA-seq dataset from our previous study that used different treatments of psychotropic drugs on the same NT2-N cell model (Bortolasci et al., 2020b). Biologically interesting modules are expected to be preserved in other samples with a homogeneous cellular population, which implies sets of genes working in concert for specific biological functions. From the results of the module preservation analysis (illustrated in Supplementary Figure S1), any module with a z-summary score higher than 10 was regarded as strongly preserved, meaning the co-expression relationships of its member genes were maintained across the two datasets. Among the 11 modules found to be strongly preserved in this independent dataset, module five was the most highly preserved module.

### Identification of Key Modules Associated With Drug Treatment (Module-Trait Analysis)

**FIGURE 2:**
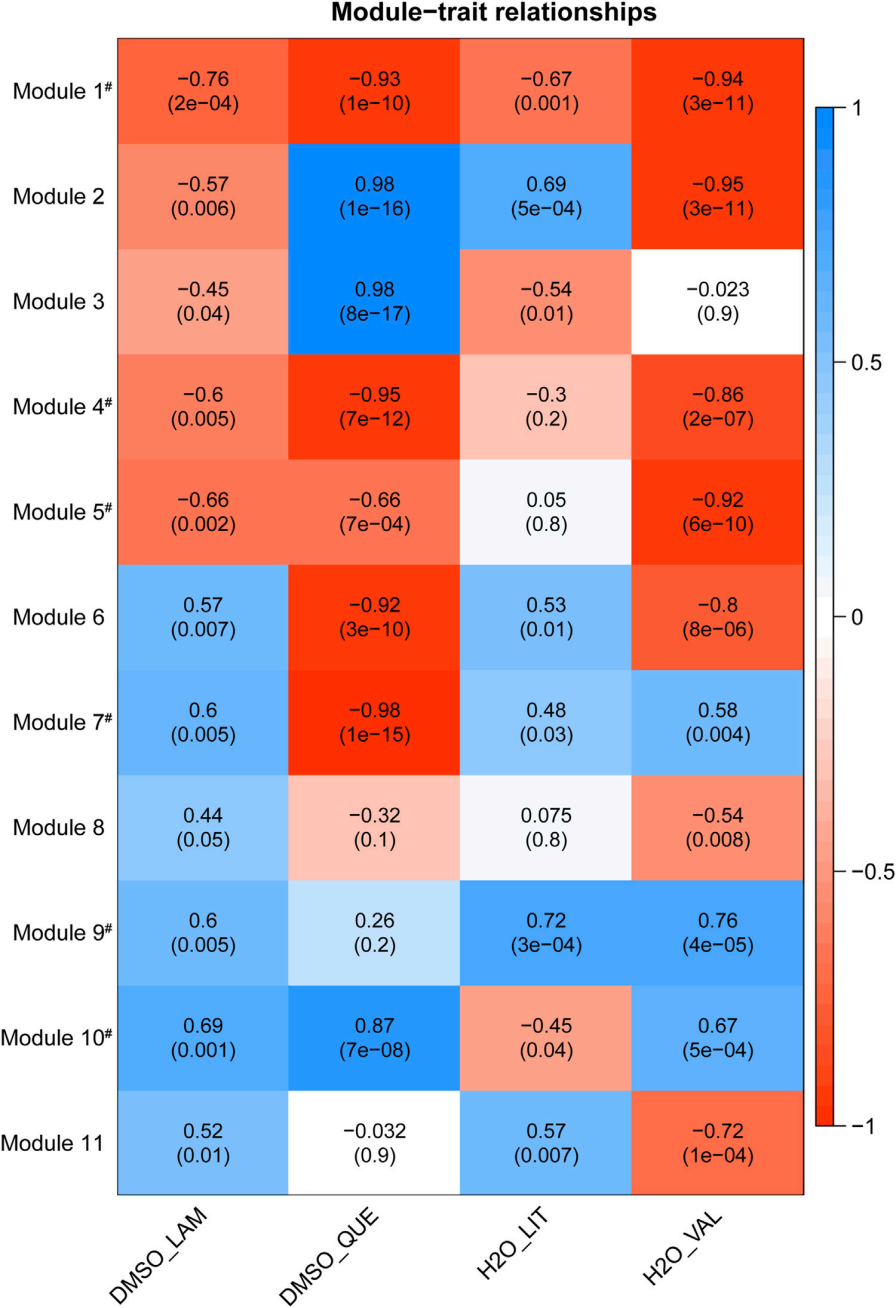
Heatmap of module-trait relationships with corresponding *p*-values between the detected modules on the *y*-axis and vehicle-drug traits on the *x*-axis. The correlation value and Benjamini–Hochberg adjusted *p*-value for each pair are labelled on the cell in the format: correlation (*p*-value). Each cell of the heatmap is coloured based on correlation between each module eigenvalue and the trait: blue is a strong positive correlation, red is a strong negative correlation, and white is little to no correlation. For example, regarding the DMSO-Lamotrigine trait, the module 5, with the negative correlation value of −0.66 and significant adjusted *p*-value of 0.002, tends to have lower overall expression (summarised as eigengene value) in lamotrigine treatment compared with the corresponding DMSO vehicle control. Abbreviation: DMSO_LAM–Lamotrigine treatment versus DMSO vehicle, DMSO_QUE–Quetiapine treatment versus DMSO vehicle, H2O_LIT–Lithium treatment versus water vehicle, H2O_VAL–Valproate treatment versus water vehicle. Modules labelled with hash (#) were coherently regulated with same directionality by at least three drugs. LncRNA-mRNA co-expression networks, identification of hub lncRNAs.

Results from the module-trait association analysis for the 11 preserved modules are shown in Figure 2 (each cell in the heatmap has a correlation value and an adjusted *p*-value). The acquired module-trait relationships enabled the evaluation of which module(s) were most affected by each drug. While the most significant module associated with each drug treatment was of interest, we cannot rule out the possibility that such an association could be accounted for by off-target effects. Hence, we further filtered modules which were coherently associated with at least three drug-vehicle pairs in a similar manner with statistical significance of adjusted *p*-value < 0.05 (correlation values can be either negative or positive, but they must share the same directionality in at least three traits), as similar effects of multiple drug treatments on one module tend to imply the common therapeutic mechanisms of the drugs rather than distinct side effects of each drug. We identified seven modules meeting such requirements (modules 1, 3, 4, 5, 7, 9, and 10—labelled with a hash symbol in Figure 2) and these modules were regarded as key modules associated with BD drug treatments. In further analyses, we focused on the key modules to find co-expressed relationships of genes belonging to each module, and which drug treatment was most strongly associated with each module.

**FIGURE 3:**
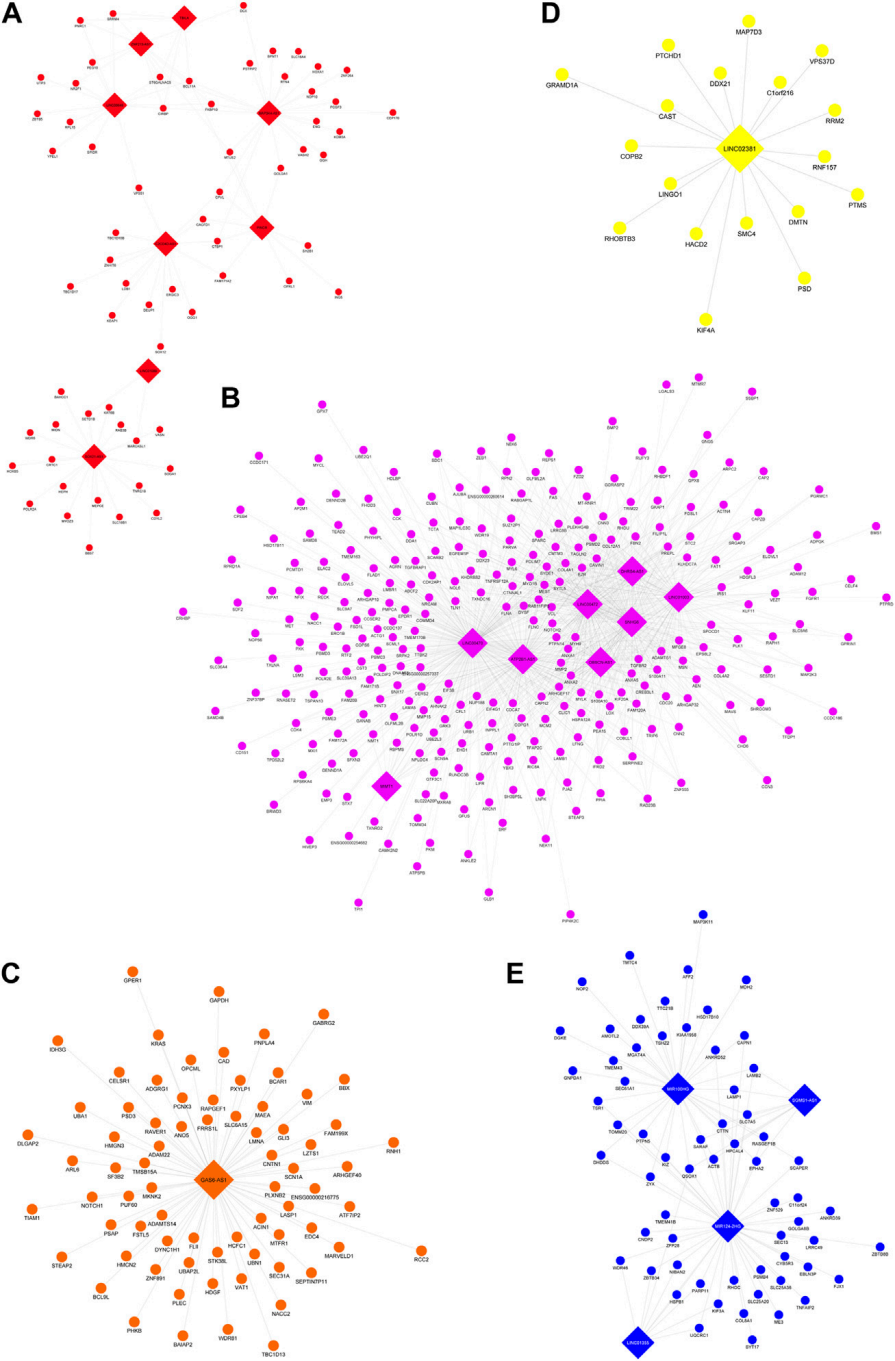
Separated lncRNA-mRNA subnetworks of five key modules. In the network, nodes representing genes and edges representing co-expression connections between them, mRNAs are circle nodes while lncRNAs are diamond shaped. Each subnetwork corresponds to a module: **(A)** Module 1 (red nodes), **(B)** Module 4 (pink nodes), **(C)** Module 5 (orange nodes), **(D)** Module 7 (yellow nodes), and **(E)** Module 10 (blue nodes).

The lncRNA-mRNA co-expression networks were constructed on the six key modules coherently regulated with the same directionality by three or more drugs to shed light on the molecular mechanism of lncRNAs that might be potential targets of drugs used to treat BD. Module nine was eliminated from this step due to the lack of lncRNA presence in the module. Hence, there were five key modules associated with the effects of BD drug treatments (i.e., modules 1, 4, 5, 7, and 10) being constructed for the lncRNA-mRNA co-expression networks. The integrated network of these modules is illustrated in Supplementary Figure S2 and separated sub-networks for single modules are shown in Figure 3.

Network statistics of lncRNAs identified from these modules are presented in Supplementary Table S3. Genes from each module were ranked by degree of centrality, which measures the number of edges (interactions) each node has. Nodes with greater importance tend to lie on multiple paths (edges) between other nodes and hence finding hub genes with high degrees of centrality can illuminate the greatest influencers of the biological networks (Mason and Verwoerd, 2007). Interestingly, the most connected lncRNAs (hub lncRNAs) identified tended to be differentially expressed in the drug treatments having highest module-trait relationships with these lncRNAs’ modules. The complementary results of differential expression analysis reinforced the role of these lncRNAs in their corresponding module: the eigen value representing a module most correlated with a certain treatment (module-trait relationship in co-expression network analysis), and hence the hub genes that have the greatest contribution to such module regulation tended to have more significant difference induced by such treatment (reflected as log fold change) than other treatments, to an extent that reached the FDR 0.05 cut-off in differential expression analysis for single genes.

### Cis- and Trans-Interactions

To explore the characteristics of regulatory relationships between lncRNAs and mRNAs, we searched for cis-targets within each module based on proximity of coding genes to transcription start sites of lncRNAs, as well as genes encoding transcription factors trans-interacting with lncRNAs based on known binding evidence from chromatin immunoprecipitation followed by sequencing (ChIP-seq) data (Supplementary Table S4). In the five lncRNA-mRNA key modules, *SETD1B* identified as potential cis-targets identified within 100 kb of *LINC01089*. We identified four lncRNAs that might potentially be regulated themselves by coding genes in the same module that are trans-acting transcription factors found nearby the promoter or enhancer region of those lncRNAs. These transcription factor-lncRNA regulation relationships were found only in several lncRNAs located in two modules, and most trans-interactions of lncRNAs-mRNAs tended to be regulated by lncRNAs rather than the other way around.

### Functional Enrichment Analysis

**FIGURE 4:**
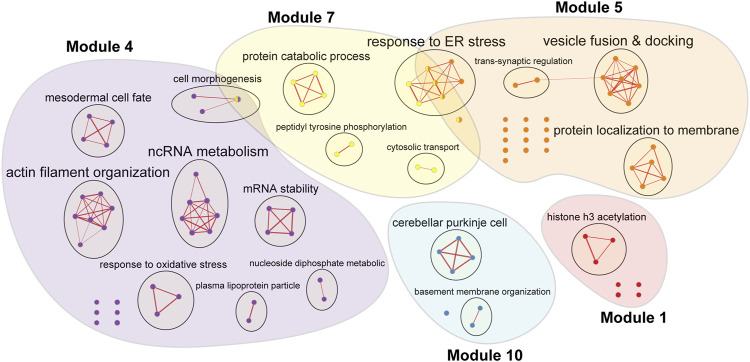
Enrichment Map of five key modules with hub lncRNAs contributing to the modular regulation induced by bipolar disorder drugs. Enriched gene sets are represented as nodes, while edges connect the similar gene sets together. Nodes are coloured by the module they enriched with the coloured patches covering all enriched gene sets by module; one node might be enriched in multiple modules. The bigger the node, the higher the number of genes found in the gene set. The thickness of each edge is proportional to the number of mutual genes between nodes. Gene sets with similar functions are clustered and labelled based on the main theme they belonged to. Abbreviation: ER–endoplasmic reticulum.

Over-representation analysis was undertaken for genes from five key modules with hub lncRNAs prominently contributing to the module regulation (i.e., modules 1, 4, 5, 7, and 10) to identify the main biological processes driven by each module (detailed results provided in Supplementary Table S5). GO terms from each module with *p*-values <0.001 were then analysed in Enrichment Map to be grouped into clusters, which highlights the major functional themes enriched in the five key modules (Figure 4).

The Enrichment Map showed a clear separation of biological functions enriched by the five key modules. Module one appears to be related to epigenetic modification via the enrichment of genes regulating histone acetylation. Module four showed enrichment in cell morphogenesis, RNA metabolism and response to oxidative stress. Module five focused on vesicle docking and fusion of trans-synaptic transmission. Module seven was enriched for peptidyl tyrosine phosphorylation and protein catabolism, which might be involved in the response to endoplasmic reticulum stress. Module 10 may regulate the formation of Purkinje cells and the basement membrane organization.

## Discussion

In the current study, multi-stepped approaches using systems biology methodology were deployed to investigate the molecular mechanisms of commonly prescribed BD drugs with a focus on lncRNAs and their potential regulatory connections with mRNAs. LncRNAs are generally expressed at lower levels than protein coding RNAs, but the cascade of regulations induced by lncRNAs may amplify the effects they produce (Ulitsky, 2016; Hu et al., 2018; Encode, 2019). However, they might be overlooked by conventional pair-wise gene expression comparisons, in which the extent of differential expression highlights significant genes for further analyses. Despite their low abundance that could be considered barely changed using conventional differential expression methods, lncRNAs can exert pronounced effects via their distinct mechanisms, e.g., one to ten molecules per cell is likely adequate for enabling cis-acting lncRNAs to affect transcription at a single locus or at several loci through either direct base-pairing with genomic DNA or recruitment of chromatin modifiers (Wu et al., 2021). Several lncRNAs were found to drive vital processes even with subtle changes such as *RepA* (X-chromosome inactivation) (Zhao et al., 2008), and *VELUCT* (lung cancer cellular viability) (Seiler et al., 2017). Since genes rarely act alone, considering them in the context of biological networks that they shared interactions with is fundamental to gain better understanding of regulatory entities such as transcription factors and lncRNAs.

Network-based analyses such as co-expression networks prioritised relationships (co-expression) rather than just the states of singular components (mean expression changes). An analysis based only on mean changes in expression could lead to an incorrect conclusion about a particular pathway’s involvement in a condition given that genes may change their partners depending on dynamic biological demands to recruit or disassociate groups of co-regulated genes for a particular task (Mentzen et al., 2009). Pathways with significant mean expression alteration but decreased co-expression could imply a change in functional assignment but do not guarantee whether genes *a priori* assumed to be in the certain biological pathways are actually dedicated to such pathways since genes could have switched roles and interacted with genes from different pathways (Mentzen et al., 2009; de la Fuente, 2010). The essential need to consider co-expression changes in addition to differential mean expression when comparing gene expression datasets has been emphasised in multiple biological contexts (Mentzen et al., 2009; de la Fuente, 2010; Savino et al., 2020; Weighill et al., 2021). For example, co-expression analyses of cancer gene expression datasets found several transcription factors known to regulate cancer development were identified as highly differentially co-expressed, despite their mean expression levels not having significant changes (de la Fuente, 2010). A proof-of-concept example from Hudson et al. showed co-expression analysis correctly identified a causal gene with a mutation between two bull varieties, whereas the gene had non-significant mean expression changes (Hudson et al., 2009).

In our analyses, co-expression network analysis utilised the whole transcriptome to offer insights on the role of lncRNAs via their connection to other biological entities such as mRNAs (Parikshak et al., 2015). Hence, such a systems biology approach is more ideal to characterise the complexity of BD as well as its treatments (Gaiteri et al., 2014). While WGCNA has been deployed in some psychiatric disorders including BD (Akula et al., 2016; Fromer et al., 2016; Kim et al., 2016; Liu et al., 2019; Zhang et al., 2021), to our knowledge, this is the first-time co-expression network analysis was used for exploring mechanism(s) of action of drugs used to treat BD.

Based on RNA-seq gene expression profiles of four BD drug treatments in NT2-N cells, we applied WGCNA to explore co-expressed genes with a focus on lncRNA-mRNA connectivity and to further identify hub lncRNAs associated with the drug treatments. In many cases, hub genes, particularly intramodular hubs, play a greater role than other network nodes in determining the network’s functionality (Horvath, 2011). Hence, identification of hub lncRNAs might illuminate major lncRNAs influencing the transcriptional regulation of their corresponding modules, which were strongly associated to a certain drug treatment. We also evaluated the type of regulatory interactions between lncRNA and mRNAs, such as potential cis mRNA targets, and trans-acting transcription factor-lncRNA relationships based on current knowledge of transcription factor binding sites. As lncRNAs might be cis- or trans-acting, while the prior can be identified based on the proximity of coding genes to the lncRNAs, pinpointing the latter is challenging due to the complex mechanisms (Statello et al., 2021). Therefore, we identified the potential transcription factors that might regulate lncRNAs, leaving the rest of lncRNA-mRNA trans-interactions more likely to be regulated by lncRNAs.

From 29 modules clustered upon the co-expression pattern of the whole dataset, we identified five key modules for lncRNA-mRNA networks as potential drivers of BD drug treatments. As the roles of lncRNAs are mainly unknown in BD, their functions can be speculated via the connected mRNAs in the modules as per the ‘guilt-by-association’ principle (van Dam et al., 2018). Such association was employed based on how genes co-expressed with one and another across all samples, with the justification that closely regulated genes are more likely to be associated with similar functions. Interestingly, our enrichment analysis showed while there was separation in functional annotations between modules, the enriched functions tended to be complementary, serving several major biological processes such as synaptic vesicle cycle, actin filament organization, endoplasmic reticulum-related functions and neurodevelopment. Findings from previous studies using co-expression network analysis for bipolar disorder have found similar processes significantly enriched by hub genes, e.g., regulation of transcription, postsynaptic density, ribosomal subunit, endocytosis (Akula et al., 2016; Liu et al., 2019), actin filament-based process, axon development (Zhang et al., 2021). Co-expression analysis on RNA-seq of whole blood from BD patients found the co-expressed modules associated to lithium usage enriched endoplasmic reticulum related functions (Krebs et al., 2020). However, it should be acknowledged that these studies mainly focused on mRNAs rather than lncRNAs for their co-expression network construction, hence the involvement of lncRNAs was not evaluated. Nevertheless, the similar enriched biological processes in BD-related phenotypes supported the findings found in our current study.

Our functional enrichment analysis on key modules highlighted their association to the synaptic vesicle cycle, in which module five was enriched for vesicle docking and fusion processes. The synaptic vesicle cycle is highly relevant to BD given that the classic pathophysiological hypothesis has been built upon dysregulation of monoamine transmission (Stahl, 2013). The expression of the hub lncRNA of module 5, *GAS6-AS1*, was upregulated by valproate–the treatment that module five correlated to the most. While the role of *GAS6-AS1* in BD is unclear, it promoted cell proliferation, migration and invasion in several cancer cell lines (Zhang et al., 2019; Li et al., 2020). Interestingly, *GAS6-AS1* was also shown to activate the PI3K/AKT pathway (Li et al., 2020), which itself plays a vital role in vesicle trafficking (Bhattacharya et al., 2016; Bilanges et al., 2019).

Targeting the endoplasmic reticulum (ER) and its related processes could be one major function regulated by BD drugs, as inferred from multiple modules in this study: response to ER stress mainly in module seven and partially in module 5. When ER stress occurs, autophagy is activated to restore cellular homeostasis, but this response is compromised in BD (Susanne et al., 2016). There is converging evidence demonstrating that lithium increases autophagy *via* inositol depletion (Bar-Yosef et al., 2019). An analysis of the lithium response gene network in BD-patient derived lymphoblastoid cell lines also identified ER stress as a major module (Breen et al., 2016). *LINC02381* was identified as the hub lncRNA in module 7. *LINC02381* has not been researched in BD previously. Evidence from gastric cancer cell lines suggested *LINC02381* can reduce Wnt pathway activity and increase apoptosis (Jafarzadeh and Soltani, 2020). Our previous study presented the downregulation of the Wnt pathway in multiple antipsychotic treatments including quetiapine, suggesting this pathway might be a common mechanism induced by different antipsychotics (Panizzutti et al., 2021). In the current study, such an effect could be partially explained by the upregulation of hub lncRNA *LINC02381* by quetiapine shown in differential expression analysis, which in turn attenuates the Wnt pathway.

Module 10 appears to contain an integrated cluster of functions related to neurodevelopment *via* its effect on Purkinje cells and the basement membrane. The basement membrane is part of the extracellular matrix system that plays a critical role in corticogenesis involving Purkinje cells in the cerebral cortex (Franco and Müller, 2011). Damage-associated molecular patterns of extracellular matrix components of the basement membrane induce an immune response suggested to be part of the pathogenesis of BD (Rege and Hodgkinson, 2013). The expression of two hub lncRNAs of module 10, *MIR124-2HG* and *MIR100HG*, were found to be differentially expressed by several drug treatments. *MIR124-2HG* is the host gene of *miR-124*, which is the most abundant brain-specific miRNA regulating neuronal differentiation during CNS development and adult neurogenesis (Sonntag et al., 2012). Moreover, *miR-124* was shown to have a pathophysiological contribution in some neuropsychiatric disorders such as Alzheimer’s disease and autism (Tabarés-Seisdedos and Rubenstein, 2009; Fang et al., 2012). *MIR100HG* (alternative name *Linc-NeD125*) was suggested in human neuroblastoma-derived cells to be specifically induced during neuronal differentiation to support cell survival (Bevilacqua et al., 2015). While *MIR100HG* encodes three miRNAs in its intron (i.e., *miR-100*, *miR-125b* and *let-7a*) (Ottaviani et al., 2018), it might work independently of the hosted microRNAs to promote the required conditions for differentiation by attenuating cell proliferation and exerting its anti-apoptotic function via the activation of BCL-2 (Bevilacqua et al., 2015). These hub genes have yet to be explained in terms of their biological functions in BD, hence more studies are needed to illuminate their involvement in the disorder.

This study is not without limitations. We used the *in vitro* model of NT2-N cells that have limited capacity to represent the disease state. In addition, the 24-h administration with a single dose for each drug for our NGS analyses limits the overall evaluation on the long-term pharmacological regulation. Finally, the analysis was focused on one dataset due to the limited availability of high-throughput expression data of BD drug responses. More representative results could be obtained from the incorporation of multiple datasets, as well as the addition of other ncRNA species such as miRNAs and siRNAs to obtain more comprehensive insights on the molecular mechanism(s) of BD drugs.

In conclusion, our study demonstrates the potential key role of lncRNAs in the regulatory effect of BD drugs via the associated lncRNA-mRNA co-expression networks. Several major processes were enriched in key modules associated with drug treatments such as synaptic vesicle cycle, cell cycle, endoplasmic reticulum-related functions and neurodevelopment. These results contribute to our understanding of the mechanisms of action of BD drugs and suggest potential novel targets for therapeutic intervention.

## Data Availability

The datasets presented in this study can be found in online repositories. The names of the repository/repositories and accession number(s) can be found below: National Center for Biotechnology Information (NCBI) BioProject database under accession number GSE197966.
