# Supplementary figures and images for: Co-Expression Networks Unveiled Long Non-Coding RNAs as Molecular Targets of Drugs Used to Treat Bipolar Disorder
Source: Front Pharmacol. 2022 Apr 8;13:873271. doi: 10.3389/fphar.2022.873271 (PMC9024411; doi:10.3389/fphar.2022.873271)

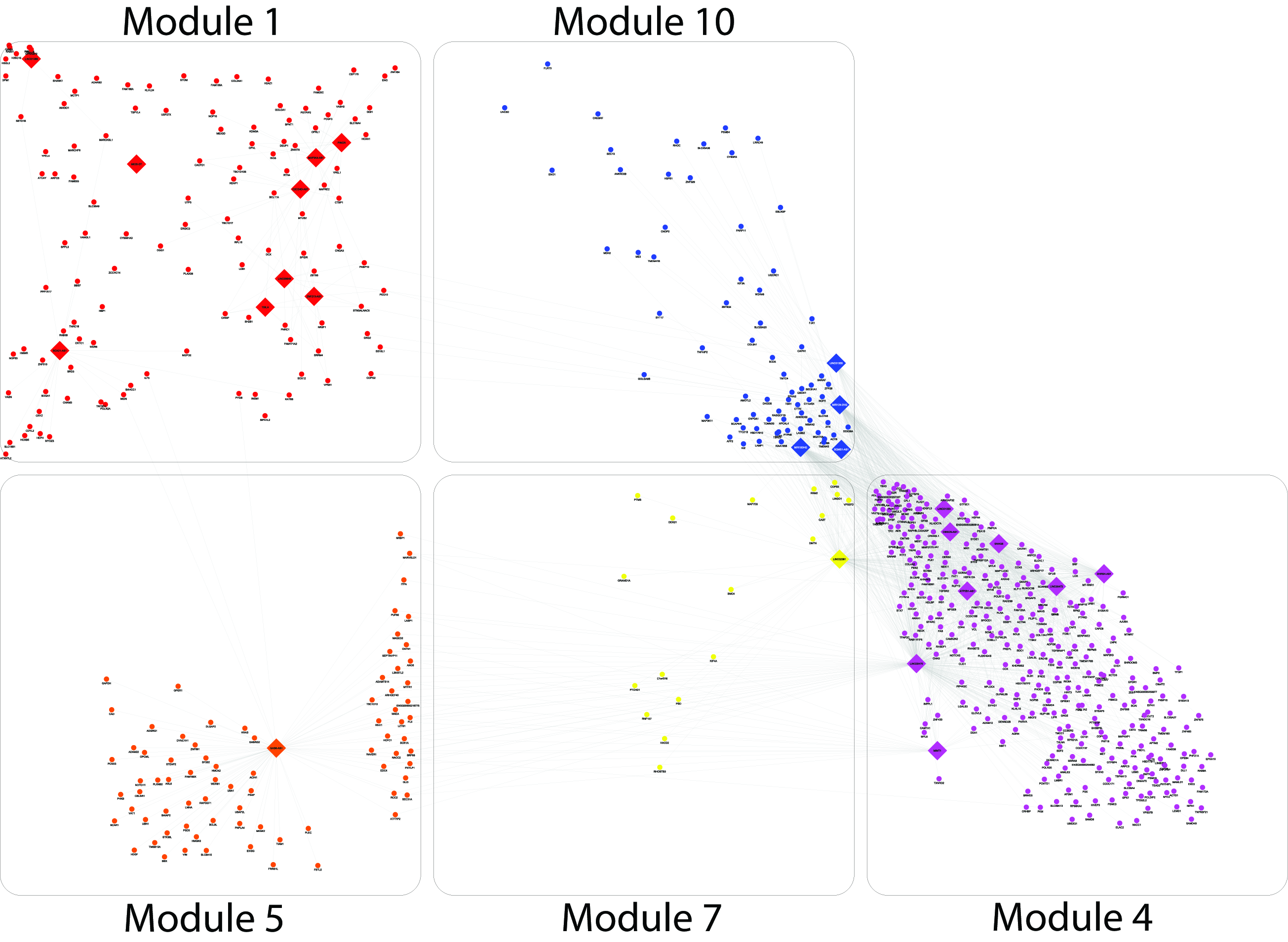

Supplement: Supplementary file 1 [file Image2.TIF]

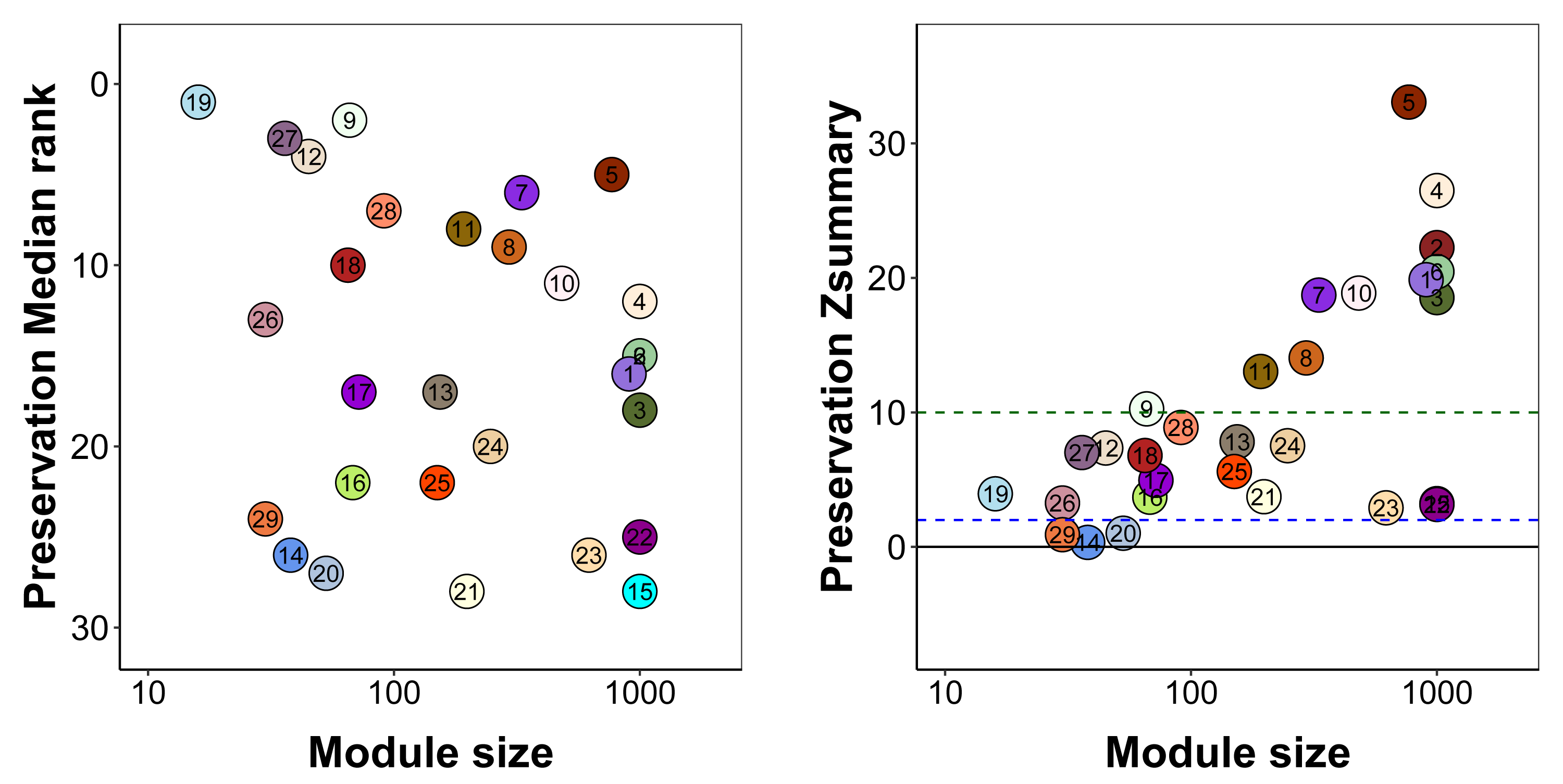

Supplement: Supplementary file 2 [file Image1.TIF]
